# Supplementary material for: MetaRibo-Seq measures translation in microbiomes
Source: Nat Commun. 2020 Jun 29;11:3268. doi: 10.1038/s41467-020-17081-z (PMC7324362; doi:10.1038/s41467-020-17081-z)
Supplement: Supplementary file 10 — Supplementary Data 7 [file 41467_2020_17081_MOESM10_ESM.zip › File2/Confidence_High_Taxonomy/386899_out.krona.html]

Javascript must be enabled to view this page.

members
magnitude
magnitudeUnassigned
count
unassigned
taxon
rank

386899\_out

4

2
superkingdom
4

1224
phylum
4

class
4
1236

91347
4
order

family
4
543

genus
4
561

4

SRS012273\_contig\_number\_26029SRS077454\_contig\_number\_8574SRS144362\_contig\_number\_23166SRS146888\_contig\_number\_15065
species
562
